# Enhancing interprofessional collaboration and interprofessional education in women’s health

**DOI:** 10.1080/10872981.2022.2107419

**Published:** 2022-08-04

**Authors:** Laura Baecher-Lind, Angela C. Fleming, Rashmi Bhargava, Susan M. Cox, Elise N. Everett, David A. Forstein, Shireen Madani Sims, Helen K. Morgan, Christopher M. Morosky, Celeste S. Royce, Tammy S. Sonn, Jill M. Sutton, Scott C. Graziano

**Affiliations:** a Department of Obstetrics and Gynecology, Tufts University School of Medicine, Boston, MA, USA; b Department of Obstetrics and Gynecology, Beaumont Hospital, Novi, MI, USA; c Department of Obstetrics and Gynecology, University of Saskatchewan College of Medicine, Regina, SK, Canada; d Department of Obstetrics and Gynecology, Dell Medical School, Austin, TX, USA; e Department of Obstetrics and Gynecology, Larner College of Medicine at the University of Vermont, Burlington, VT, Canada; f Department of Obstetrics and Gynecology, Rocky Vista College of Osteopathic Medicine, Parker, CO, USA; g Department of Obstetrics and Gynecology, University of Florida College of Medicine, Gainesville, FL, USA; h Department of Obstetrics and Gynecology, University of Michigan Medical School, Ann Arbor, MI, USA; i Department of Obstetrics and Gynecology, University of Connecticut School of Medicine, Farmington, CT, USA; j Department of Obstetrics and Gynecology, Beth Israel Deaconess Medical Center, Boston, MA, USA; k Department of Obstetrics, Gynecology, and Reproductive Biology, Harvard Medical School, Boston, MA, USA; l Department of Obstetrics and Gynecology, Washington University School of Medicine, St. Louis, MO, USA; m Department of Obstetrics and Gynecology, Brody School of Medicine at East Carolina University, Greenville, NC, USA; n Department of Obstetrics and Gynecology, Loyola University Medical Center, Hines, IL, USA

**Keywords:** Accreditation, clinical competence, communication skills, competency-based education, curriculum development, health-care professionals, interdisciplinary, medical education

## Abstract

This article is from the ‘To The Point’ series from the Association of Professors of Gynecology and Obstetrics Undergraduate Medical Education Committee. The purpose of this review is to provide an understanding of the differing yet complementary nature of interprofessional collaboration and interprofessional education as well as their importance to the specialty of Obstetrics and Gynecology. We provide a historical perspective of how interprofessional collaboration and interprofessional education have become key aspects of clinical and educational programs, enhancing both patient care and learner development. Opportunities to incorporate interprofessional education within women’s health educational programs across organizations are suggested. This is a resource for medical educators, learners, and practicing clinicians from any field of medicine or any health-care profession.

Health-care teams have faced enormous challenges over the past several years, navigating a pandemic while also continuing to provide routine and preventive health care, striving to mitigate disparities and racism, training our next generation of health-care providers, and combatting chronic public health crises such as violence and mental health in the USA. Each of these tasks are Herculean and require collective ingenuity and efforts across specialties. Taken alone, the COVID-19 pandemic has required collaboration between physicians, public health experts, respiratory technicians, nurses, environmental services, among others. Without the complementary yet distinct skills and expertise of each of these disciplines, more profound morbidity and mortality may have occurred [1]. Put simply, the health-care challenges that we face cannot be surmounted by physicians alone. The complexity of health and health care in the 21st century demands increasingly coordinated and agile collaboration among varied health-care professionals [2]. Medical educators must ensure future physicians are trained experts at engaging with non-physician health-care professionals in a respectful, productive, and collaborative manner in order to manage day-to-day health care as well as to solve the challenges ahead.

## Collaboration versus Education

**Figure 1.**
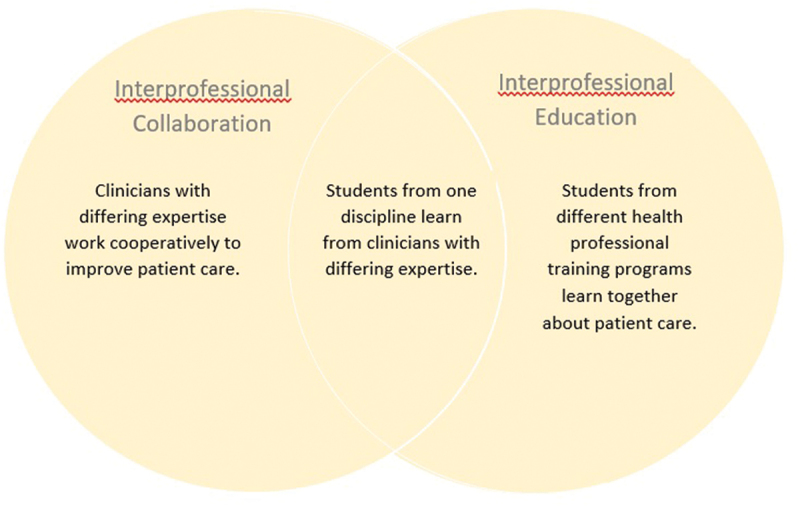
Relationship between interprofessional collaboration and interprofessional education with examples.

The terms Interprofessional Collaboration (IPC) and Interprofessional Education (IPE) are often used interchangeably; however, they refer to distinct yet overlapping entities (Figure 1) [3]. Interprofessional education (IPE) refers to the co-education of learners from different disciplines, such as pharmacy and medicine, learning alongside one another as well as about each other’s professions in a classroom or workplace learning environment. A common set of learning objectives for all students engaging in interprofessional education – regardless of discipline – has been endorsed by over 60 professional societies and includes four core competencies related to the value of collaboration, roles and responsibilities, communication, and teamwork [4]. Medical students often work alongside physician assistants, advanced practice nurses, and other students during their clinical education. Learners from different schools typically have distinct learning objectives and educational goals reflective of their future discipline and learn not only the patient care-related material alongside one another but also how the roles and perspectives of the other discipline complement patient care to achieve the core interprofessional education competencies.

In comparison, interprofessional collaboration (IPC) refers more specifically to the workplace rather than the educational environment and focuses on health-care professionals from different disciplines working cooperatively to optimize patient care. IPC occurs when clinicians from different backgrounds – such as physical therapy, physicians, and audiologists – work together using their distinct yet complementary skills and expertise to provide the highest quality of care to patients, families, and communities [5]. Substantial overlap between IPE and IPC exists given the inclusion of students into multidisciplinary teams on a regular basis in the clinical setting. As such, learners can achieve similar educational goals through IPC experiences. The communication, perspective enhancing, and collaborative skills that are the objectives of IPE may be more readily achieved through IPC opportunities already existent in health-care settings [4]. For example, arranging for case-based or patient simulation learning experiences that are mutually beneficial for students from differing vocations – such as a birthing simulation experience for medical and midwifery students – are often time-consuming and challenging to implement [5]. Instead, having a medical student spend a Labor and Delivery shift with a midwife rather than the obstetrics team, and having a midwifery student work with the obstetrics team can be much simpler to implement and potentially more effective to achieving educational goals.

An emphasis on IPE should lead to improved IPC. That is, an educational environment that respects and encourages IPE should produce health-care professionals that are more agile in involving and respecting one another’s contributions. Conversely, an educational environment where learners remain siloed, surrounded only by students from their own discipline and taught exclusively by faculty from their own domain, might be less likely to understand, involve, and respect the contributions of other professionals involved in patient care.

Both IPC and IPE have been increasing in prominence in health-care and medical education over the past several decades. Prior to 1999, IPE and IPC were considered fringe concepts in higher medical education and health-care settings. In 1999, *To Err is Human* was published by the then Institute of Medicine which revealed the frequency of medical errors and recommended improved collaboration between health-care professionals – called ‘caregiving microsystems’ in the report – to improve patient safety [6]. These caregiving microsystems are what we would call IPC today. With the increasing emphasis on IPC in health-care workplaces, educators developed the concept of IPE to train students to be able to interact in an increasingly collaborative health-care environment. In 2010, the World Health Organization published a framework for IPE for global medical education [7]. This was followed in the US by the release of recommendations from a collaboration of the six main educational organizations in health-care education – specifically, American Association of Colleges of Nursing, American Association of Colleges of Osteopathic Medicine, American Association of Colleges of Pharmacy, American Dental Education Association, Association of American Medical Colleges, and Association of Schools of Public Health – which hallmarked the widespread introduction of IPE into medical education curricula at medical schools nationwide [8].

As of 2013, the Liaison Committee on Medical Education (LCME) required medical schools to demonstrate that students are being prepared to function collaboratively on health-care teams that include other professionals. This requirement remains a part of LCME accreditation currently represented as Standard 6.7, which requires that medical students ‘have opportunities to learn in academic environments that permit interaction with students enrolled in other health professions, graduate, and professional degree programs, and in clinical environments that provide opportunities for interaction with physicians in graduate medical education programs and in continuing medical education programs’, and Standard 7.9, which requires that medical schools ensure that students are prepared to ‘function collaboratively on health-care teams that include health professionals from other disciplines as they provide coordinated services to patients’ [9].

## IPE and IPC in obstetrics and Gynecology

Obstetrics and Gynecology has long been a collaborative specialty. Even well into the 20th century, women sought care from traditional birth attendants for expertise in pregnancy and childbirth rather than from a physician. In the 1940s, nurse midwifery was promoted by public health nurses, social reformers, and obstetricians in order to reduce maternal morbidity and mortality [10]. Expertise in pregnancy and childbirth is now shared between obstetricians and other health-care providers including nurse midwives, family medicine physicians, women’s health nurse practitioners, physician assistants, and doulas. Nearly 13% of women in the USA choose a midwife rather than an obstetrician for their care [11]. Women receiving care with midwives experience fewer interventions in labor and have reduced risks of cesarean section or operative vaginal delivery [12]. It is recognized that increasing access to and learning best practices from nurse midwifery may be a primary strategy to continuing to reduce maternal morbidity and mortality in the USA [11–13]. Team-training, a form of interprofessional education, has been shown to reduce rates of adverse obstetric events including return to the operating room and birth injury [14].

Gynecologic subspecialties also commonly rely on non-physician health-care professionals to deliver care and improve patient outcomes and safety. In Gynecologic Oncology, nurse navigators improve coordination of care and are associated with increased patient satisfaction and reduced anxiety [15]. Reproductive Endocrinology regularly incorporates psychological assessment and support for patients undergoing evaluation and treatment for infertility which is associated with reduced anxiety and improved success with fertility treatments [16,17]. Urogynecologists routinely incorporate pelvic floor physical therapy into treatment planning for incontinence and prolapse which improves successful treatment and patient satisfaction[18].

Other non-physician health-care professionals have substantial roles in improving outcomes for Obstetrics and Gynecology patients across subspecialties. Involving pharmacists in bedside rounds reduces the risk of medication errors by two-thirds [19]. When physical therapists regularly participate in discharge planning, readmission rates are decreased more than twofold [20]. Routinely involving genetic counselors across disciplines is associated with increased patient satisfaction, a greater sense of control, and positive health behaviors [21]. Patients receiving pastoral care report greater sense of peace and reduced anxiety about their prognoses [22].

Interprofessional education is paramount to ensuring a future workforce that is receptive to the knowledge, perspectives, and expertise of other disciplines. Working effectively in interdisciplinary teams has been identified as the single-most important skill for incoming residents and is a required component of all residency training programs [23,24]. Evidence indicates that IPE activities improve learners’ attitudes towards other members of interdisciplinary teams and enhance communication and shared problem-solving among interdisciplinary team members [25]. Given the clear patient care and population health benefits that interdisciplinary teams offer, the evidence supporting the efficacy of IPE on achieving a physician workforce more proficient in interdisciplinary teamwork and problem-solving, and the challenges inherent in health care in the 21^st^ century, educators should strive to incorporate IPE into medical education at every opportunity.

## IPE in ObGyn clinical learning environments

As pioneers in IPC, Obstetrician-Gynecologists are well positioned to be leaders in IPE. Opportunities for direct education of medical students by non-physician health-care professionals, and for collaborative education for learners from various disciplines, may be found throughout the Obstetrics and Gynecology Clerkship. Table 1 provides examples of interprofessional collaboration in Obstetrics and Gynecology subspecialties and potential opportunities for associated IPE that can be incorporated into clinical education curricula.

**Table 1.** Opportunities for interprofessional collaboration and education within Obstetrics and Gynecology learning environments.

Clinical learning environment	Interprofessional collaboration (IPC)	Opportunity for interprofessional education (IPE)
Obstetrics – outpatient	Social workers (SW)	Spend ½ day with perinatal SW
	Ultrasound technologists	Spend ½ day with OB US technician
	Genetic counselors	Spend ½ day with GC; create family tree
	Lactation counselors (LC)	Participate in education session with LC
	Nutrition	Sit in on nutrition counseling session
	Pelvic Floor Physical Therapist (PT)	Spend ½ day with Pelvic Floor PT
Obstetrics – inpatient	Certified nurse midwifes (CNM)	Participate in labor of 1+ CNM patients
	Labor and delivery nurses	Shadow L&D nurse for one shift
	Physician assistants	Postpartum round with PA
Gynecology (all)	Pharmacists	Invite pharmacists to round daily
	Interpreter services	Shadow interpreter services for ½ day
Gynecologic Oncology	Physical therapists	Shadow PT on 1+ shared patients
	Occupational therapists (OT)	Shadow OT on 1+ shared patients
	Respiratory therapists (RT)	Shadow RT on 1+ shared patients
	Social workers	Spend ½ day with social worker
	Nutrition	Sit in on nutrition counseling session
	Pastoral care	Attend family meeting; shadow on rounds
	Genetic counselors (GC)	Spend ½ day with GC; create family tree
	Chemotherapy nurses	Shadow chemo nurse for one shift
	Palliative care	Participate in palliative care consult
Urogynecology	Pelvic floor physical therapists	Designated session with pelvic floor PT
	Nurse practitioners (NP)	Designated session with NP
Reproductive Endocrinology	Psychologists	Sit in on infertility-related counseling
	Embryologists	Shadow during infertility care

Medical students often share clinical learning environments with students from physician assistant, nursing, nurse practitioner, and genetic counseling programs, among others. To encourage IPE, educators may consider placing students with complementary yet differing educators rather than adhering to siloed educational spheres [5]. For example, a genetic counseling student may benefit from spending time with sonographers; a sonography student may benefit from spending time in a Maternal-Fetal-Medicine clinic session; and a medical student may benefit from spending time with a genetic counselor. Such an approach is an example of IPC itself, as this would require coordination across and among various educational leaders. This approach may not only complement educational goals but may also increase collaboration that translates across other domains such as patient care, clinical research, or quality and safety initiatives. Medical education leaders should create opportunities for students from other disciplines to learn from physicians and concurrently develop relationships to allow opportunities for medical students to learn from non-physician professionals in return.

Similarly, both IPC and IPE may be enhanced when students participate in interdisciplinary activities already embedded into clinical settings. For instance, interdisciplinary team rounding on antepartum patients may involve a Maternal Fetal Medicine physician, a pharmacist, and a nurse. Involving a student from each discipline to accompany each clinician – a medical student, pharmacy student, and nursing student, in this example – can complement both discipline-specific learning objectives alongside shared interprofessionalism competencies[4]. Opportunities for similar IPE exist in any situation where physicians regularly engage with non-physician health-care professionals, including subspecialty interdisciplinary rounds, consult particularly with non-physician professionals embedded in the workplace, in management of depression or other mental health concerns, or discharge planning rounds.

Educational leaders should be familiar with where these opportunities already exist in their organization, where they might be developed, and strive to incorporate learners in these activities wherever possible. The opportunities listed in Table 1 are not exhaustive; organizations may have additional or unique IPE opportunities depending upon the patient populations and services offered within that organization. Similarly, every situation of IPC does not need to be utilized as an educational opportunity. Educational leaders hoping to increase IPE within their curriculum should identify opportunities that are most easily and reliably integrated into their program in order to be self-sustaining and available to all learners. To this end, securing buy-in with key stakeholders from the collaborating profession is crucial. For example, if an educational leader hopes to incorporate a medical student working with a Labor and Delivery nurse for a shift, obtaining support from perinatal nursing and nursing leadership will be paramount. Educational leaders should monitor the impact of the IPE experience regularly with both learners and collaborators, to ensure that the experience meets educational objectives and clinical goals on an ongoing basis. The impact of IPE experiences typically involves assessment of learners’ attitudes, perspectives, and communication skills. These may be self-assessed by the learner and/or by the non-physician health-care professionals or learners within a particular IPE experience [26,27].

## Discussion

Working effectively, productively, and respectfully with diverse team members is crucial for health-care delivery across settings and in both routine as well as pandemic-related care. Medical educators and leaders must ensure our learners – the workforce of our future – are capable and comfortable collaborating with other health-care professionals in order to prepare our students for success, both personally and for the tremendous challenges that lie ahead in health care and population health.
